# Assessing the importance of interspecific interactions in the evolution of microbial communities

**DOI:** 10.1002/ece3.9494

**Published:** 2022-11-15

**Authors:** Tiffany Raynaud, Manuel Blouin, Marion Devers‐Lamrani, Dominique Garmyn, Aymé Spor

**Affiliations:** ^1^ Agroécologie, Institut Agro, INRAE Univ. Bourgogne, Univ. Bourgogne Franche‐Comté Dijon France

**Keywords:** abiotic environment, experimental evolution, interspecific interactions, productivity, synthetic bacterial communities

## Abstract

Interspecific interactions play an important role in the establishment of a community phenotype. Furthermore, the evolution of a community can both occur through an independent evolution of the species composing the community and the interactions among them. In this study, we investigated how important the evolution of interspecific interactions was in the evolutionary response of eight two‐bacterial species communities regarding productivity. We found evidence for an evolution of the interactions in half of the studied communities, which gave rise to a mean change of 15% in community productivity as compared to what was expected from the individual responses. Even when the interactions did not evolve themselves, they influenced the evolutionary responses of the bacterial strains within the communities, which further affected community response. We found that evolution within a community often promoted the adaptation of the bacterial strains to the abiotic environment, especially for the dominant strain in a community. Overall, this study suggested that the evolution of the interspecific interactions was frequent and that it could increase community response to evolution.

## Graphical abstract

The evolution of a community can not only occur through an evolution of the species composing the community but also of the interactions among them. In this study, we found evidence for an evolution of the interactions in half of the studied bacterial communities, which gave rise to a mean change of 15% in community productivity as compared to what was expected from the individual responses. It suggested that the evolution of the interspecific interactions was frequent and played a role in community evolutionary response.

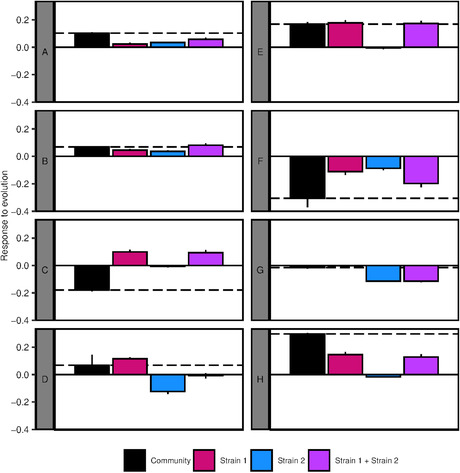

## 1 INTRODUCTION

What makes a community is the existence of interactions between the species composing the community (Liautaud et al., 2019; Whitham et al., 2020). These interspecific interactions give rise to emergent properties at the community level, i.e., characteristics that are not predictable from the sum of the properties of the component species (Guo & Boedicker, 2016; Madsen et al., 2018). Thus, it can be relevant to consider the phenotype of a community as a whole. Moreover, it is increasingly recognized that community phenotype can respond to evolution (Whitham et al., 2006). From a theoretical standpoint, it is accepted that microbial community evolution can occur through genetic changes in the community members (e.g., through mutations, horizontal gene transfer, and gene loss; Barraclough, 2015; Gorter et al., 2020). Furthermore, as well as the interspecific interactions are involved in the establishment of community phenotype, there is also evidence that they can contribute to the evolution of this phenotype. This has been investigated in the field of artificial selection at the community level through modeling (Williams & Lenton, 2007) and experimental approaches on communities made of two beetle species (Goodnight, 1990). Both approaches highlighted that independent genetic changes in the species within a community are not always sufficient to explain the observed response of the community to evolution by selection. It suggested that the interspecific interactions, whether they be under the genetic or epigenetic influence, can be involved in community evolution.

In parallel, other studies provided detailed assessments of the evolution of interspecific interactions in synthetic microbial communities. It has been shown that, in a two‐species bacterial community, a mutation in one of the two strains induced a shift from a commensal interaction to a more exploitative one (Hansen et al., 2007). This shift in the interaction occurred after 5 days of experimental evolution and gave rise to enhanced productivity at the community level. Thus, the interspecific interactions can evolve through the evolution of one of the community members (e.g., a genetic change in one of the species that induces a change in the interaction with the other species). Another way for the interactions to evolve is through the evolution of multiple species in a community. As an example, an experimental study showed that, in a four‐species bacterial community, changes in resource use in the four species when experimentally evolved together reduced the occurrence of negative interspecific interactions (Lawrence et al., 2012). It was associated with higher productivity at the community level than that of a community that was built from the four species evolved in isolation. Finally, interspecific interactions can also evolve through the evolution of several species in a community as a result of coevolution, i.e., reciprocal adaptive changes in two populations or species (Brockhurst & Koskella, 2013; Janzen, 1980).

An additional level of complexity emerges from a possible influence of the abiotic environment on the evolution of interspecific interactions in a community. For example, in bacterial communities, the evolution of interspecific interactions can be promoted by a structured environment, allowing the formation of biofilm, as compared to a homogeneous environment (Hansen et al., 2007). It has also been shown that whether or not interactions are involved in a bacterial community evolutionary response can depend on the resources or on the pH of the culture medium (Fiegna et al., 2015). Interestingly, in the study of Fiegna et al. (2015), community productivity increased as compared to the ancestral community only when the interactions were involved in the community response to evolution. To go further, the influence of the abiotic environment on the evolution of interspecific interactions can occur through niche construction (Matthews et al., 2014). This occurs when the abiotic environment is modified by a species, which in turn influences the evolution of other species in the community. For example, it has been shown that the pairwise interaction between a bacterial population and a yeast shifted from commensalism to amensalism and then to antagonism when the environment started to be changed by the yeast. Indeed, the excretion of a bacterial growth inhibitor promoted the evolution of resistance in the bacterial population, which lowered the fitness of the yeast (Andrade‐Domínguez et al., 2014). Thus, eco‐evolutionary feedbacks are also involved in the evolution of interspecific interactions and of the communities.

There are many studies that illustrate well the evolution of interspecific interactions, the question is not *whether the interactions can evolve* but *how important is the evolution of the interactions in the communities* (Gorter et al., 2020). In this study, we aimed at providing an insight into how frequently the evolution of interspecific interactions was involved in the evolution of community phenotype. Following a five‐month experimental evolution of synthetic bacterial communities (Raynaud et al., 2022), we re‐isolated eight pairs of strains that evolved within different communities. We assessed the bacterial strain and community (i.e., co‐cultures) phenotypes by measuring the optical density as a proxy of productivity. We compared the phenotypes after the experimental evolution to the ancestral phenotypes (i.e., before experimental evolution) and to the phenotypes obtained by assembling the same strains evolved in isolation to discuss the evolution of interactions. We hypothesized that: (i) the interspecific interactions played a role in the evolution of community phenotype (i.e., the phenotype of the evolved community would be different from this of a community reconstructed from strains that evolved in isolation); (ii) this role occurred through an evolution of the interactions themselves (i.e., the evolutionary response of the community would not be predictable from the separate evolutionary responses of the strains composing the community); (iii) the evolution of community phenotype depended on the abiotic environment. To verify this third hypothesis, we assessed the phenotype of the strains and communities after experimental evolution in a second abiotic environment in order to discuss the adaptation to the abiotic conditions of the experimental evolution. This study evidenced that the interspecific interactions were often involved in the evolution of the bacterial communities and that their evolution contributed to changes in community productivity.

## 2 MATERIALS AND METHODS

### 2.1 Origin of the studied communities

The eight two‐strain communities studied in this experiment stemmed from an experimental evolution procedure in which bacterial strains (= monocultures) and communities (= co‐cultures) were grown for 5 months with a serial transfer every 3.5 days. This experiment involved 18 laboratory strains that were used to create communities differing in their initial richness levels (see Raynaud et al., 2022). During the experimental evolution, the strains and communities were grown in sterile 2 ml deep‐well plates (Porvair Sciences, Wrexham, UK) filled with 1 ml of a mix of 1:5 lysogeny broth (LB) and 1:5 tryptic soy broth (TSB), hereafter called EE medium for Experimental Evolution, and placed at 28°C without shaking. An optical density (OD) measurement (600 nm) was performed at each serial transfer (i.e., measurement of the light scattered by the bacterial cells in suspension as a proxy of productivity) and the transfer occurred following two treatments: artificial selection (where the transferred culture was the one with the highest OD among 10) and no artificial selection (where the culture was transferred whatever its OD). The strains and communities were stored at −80°C in 30% glycerol before the experimental evolution (ancestors) and after the experimental evolution (evolved strains and communities). In the first step of isolation, all of the 2‐species communities (six), both under artificial selection and no artificial selection (see Raynaud et al., 2022), were considered for being analyzed in the present study. In the second step, all of the 4‐species communities (six) either under artificial selection or under no artificial selection were also considered to complete the experimental design. The pairs of strains that were finally included in the experiment are presented in Table 1 and responded to the following criteria: successful isolation of the strains from the evolved community and availability of the corresponding strains evolved in isolation. The resulting experimental design was not suitable to test neither for an effect of the initial richness level of the native community (two or four strains) nor for an effect of the selection regime applied to the native community (artificial selection or no artificial selection).

**TABLE 1:** Two‐strain communities studied in the experiment

Community identifier	Strains		Initial richness level of the native community	Selection regime applied to the native community
A	1	*Variovorax* sp. 38R	2 strains	No artificial selection
	2	*Pseudopedobacter saltens* DSM12145		
B	1	*Variovorax* sp. 38R	4 strains	No artificial selection
	2	*Pseudopedobacter saltens* DSM12145		
C	1	*Pseudomonas knackmussii* DSM6978	4 strains	No artificial selection
	2	*Variovorax* sp. 38R		
D	1	*Pseudomonas* sp. ADPe	2 strains	No artificial selection
	2	*Escherichia coli* WA803		
E	1	*Pseudomonas knackmussii* DSM6978	4 strains	No artificial selection
	2	*Pseudopedobacter saltens* DSM12145		
F	1	*Pseudomonas* sp. ADP3	4 strains	No artificial selection
	2	*Escherichia coli* K12		
G	1	*Escherichia coli* WA803	4 strains	Artificial selection
	2	*Agrobacterium* sp. 9023		
H	1	*Pseudomonas* sp. ADPe	2 strains	Artificial selection
	2	*Escherichia coli* WA803		

*Note*: Some of the pairs of strains evolved in the absence of other strains (i.e., in two‐strain native communities), whereas other pairs evolved in the presence of other strains (i.e., in four‐strain native communities), this is specified in the column “Initial richness level of the native community”. Some of the native communities evolved under artificial selection whereas others evolved under “no artificial selection” (i.e., natural selection only), this is specified in the column “Selection regime applied to the native community”. In each community, strain 1 is the most productive of the two strains (highest OD) and strain 2 is the least productive one (lowest OD).

### 2.2 Isolation of the strains from the evolved communities

To isolate the strains that evolved in communities, we revived the evolved communities from glycerol stocks by growing them on agar plates (EE medium) by streaking. After 72 h of growth at 28°C, we picked the colonies of differing morphologies and placed them on new separated agar plates by streaking. After a new cycle of growth, one colony per plate was picked and placed in 200 μl of 0.9% NaCl, and 100 μl of this suspension was plated on an agar plate with glass beads. At this step, 2 μl of suspension was used to perform a PCR for the identification of the strains (see below). After a new cycle of growth, several colonies were picked on each plate and put in 20 ml of EE medium in a flask (48 h, 120 rpm). 800 μl of suspension were then stored at −80°C in 800 μl of 60% glycerol. As these isolation steps required four growth cycles during which evolution could act, we also performed these four growth cycles in the same conditions for the corresponding ancestral and evolved in isolation strains.

### 2.3 Identification of the strains

A PCR targeting 16 S rRNA gene with the primers 27F/1492R (Miller et al., 2013) was performed for each strain isolated from the evolved communities. Digestion of the PCR products was then performed with the *Alu*I restriction enzyme and followed by electrophoresis for the identification of the strains at the genus level. For the genera that were represented by several strains in our experiment (i.e., *Pseudomonas* and *Escherichia*), we performed further analyses for identification at the strain level. We used data from *gyrB* sequencing at the community level (Raynaud et al., 2022) to determine which *Pseudomonas* strain was present in the community and coupled it with analyses at the strain level for formal identification. The different strains were identified based on the presence or not of *atzD* gene (assessed by PCR) and the resistance or not to nalidixic acid and amoxicillin (assessed by growing the strains on agar plates containing a mix of the two antibiotics at a final concentration of 100 μg ml^−1^). *Escherichia coli* K12 and *Escherichia coli* WA803 were identified based on their ability to do or not lactose fermentation (which was assessed by growing the strains on agar plates on Drigalski agar medium).

### 2.4 Evolutionary history treatments

**FIGURE 1:**
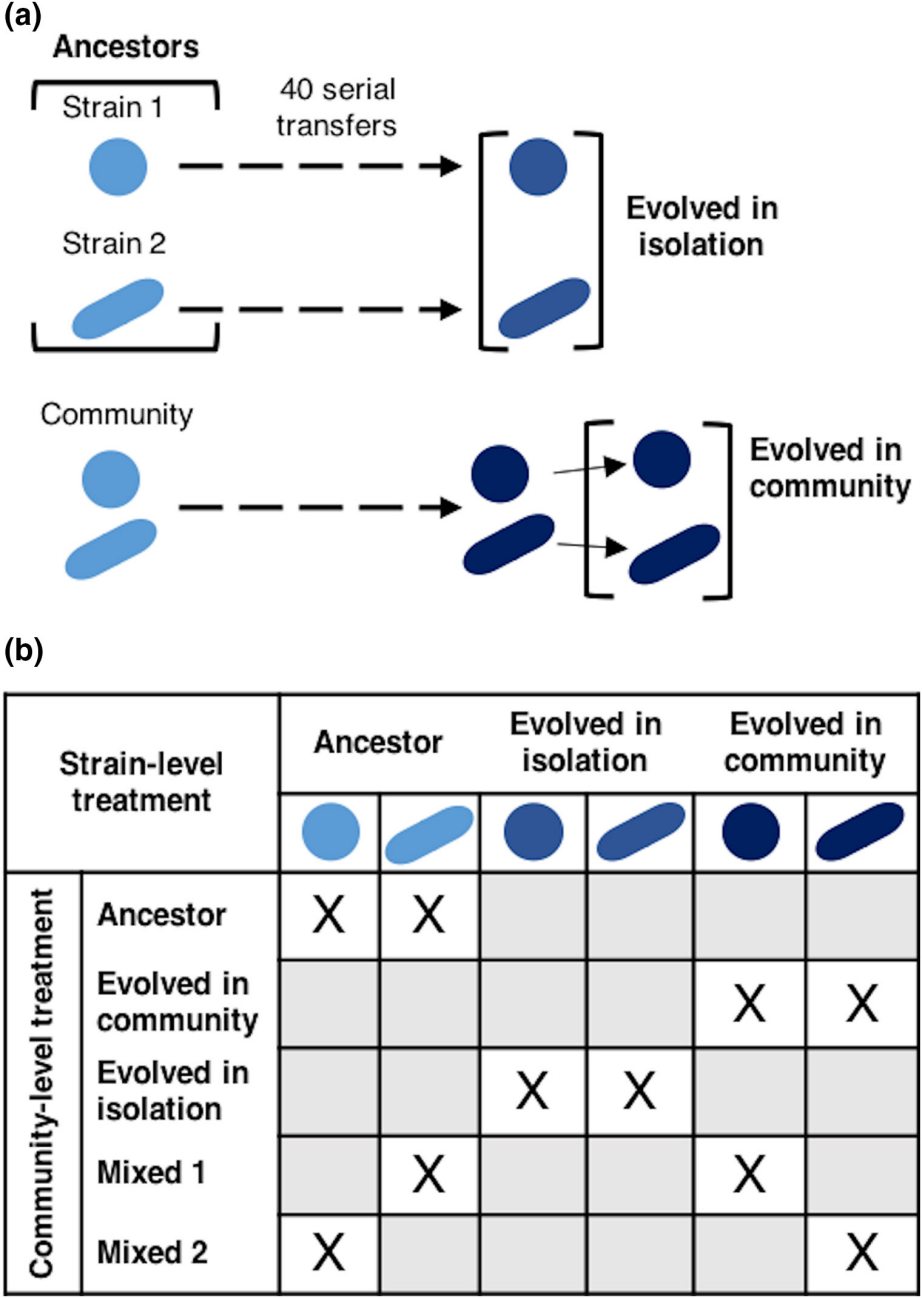
Experimental design. (a) Each bacterial strain was previously experimentally evolved in isolation and as a member of a community (Raynaud et al., 2022). At the end of this experimental evolution, the strains were isolated from the community in which they evolved. (b) From the strains, different communities were built: ancestor (co‐culture of two ancestral strains), evolved in community (co‐culture of two strains that evolved together), evolved in isolation (co‐culture of two strains that evolved in isolation), mixed 1 (co‐culture of one ancestral strain and one strain evolved in community), and mixed 2 (co‐culture of one ancestral strain and one strain evolved in community conversely to mixed 1).

Each of the two strains of a community (eight in total, hereafter identified as communities A to H; Table 1) was grown in its ancestral version (i.e., before experimental evolution), in its “evolved in isolation” version (i.e., after experimental evolution as an isolated strain), and in its “evolved in community” version (i.e., after experimental evolution within a community). It resulted in six treatments (two strains and three evolutionary histories per strain; Figure 1a). Within each community (i.e., co‐culture of two strains), the most productive (highest OD_600nm_ at 3.5 days) of the two ancestral strains were referred to as “strain 1” and the least productive was referred to as “strain 2”. In addition, each community was grown in its ancestral version (i.e., co‐culture of the two ancestral strains), in its “evolved in isolation” version (i.e., co‐culture of the two strains that evolved in isolation), and in its “evolved in community” version (i.e., co‐culture of the two strains that evolved together within a community). Two treatments mixing ancestral strains and strains evolved in community were also included: mixed community 1 (i.e., co‐culture of strain 1 evolved in community and ancestral strain 2) and mixed community 2 (i.e., co‐culture of strain 2 evolved in community and ancestral strain 1). It resulted in five treatments at the community level (Figure 1b) plus the six treatments at the strain level (Figure 1a).

### 2.5 Community construction, growth conditions and phenotype assessment

Before the start of the experiment, each strain was revived from the glycerol stock and grown in 20 ml of EE medium in a flask (48 h, 28°C, 110 rpm). The OD (600 nm) of the suspensions was measured (200 μl per well in a microplate, Infinite M200 PRO, Tecan, Männedorf, Switzerland) and the suspensions were diluted to a final OD of 0.002 in EE. The eight two‐strain communities were built by mixing an equivalent volume of each of the suspensions of the required strains. Then, two plates per community were inoculated with the suspensions at OD 0.002: a 2 ml deep‐well plate (Porvair Sciences, Wrexham, UK, 1 ml of suspension per well, eight replicates per treatment) and a honeycomb plate (Thermo Fisher Scientific, Waltham, Massachusetts, USA; 400 μl of suspension per well, eight replicates per treatment). The growth conditions in deep‐well plates were: 28°C, no shaking; the OD was measured after 3.5 days of growth by homogenizing the well content, pipetting 200 μl of suspension, and transferring it into a new plate for OD measurement at 600 nm (Infinite M200 PRO). These growth conditions were identical to the growth conditions of the experimental evolution, hereafter we refer to these conditions as “environment 1”. We wanted to test whether the evolution of the community phenotype depended on the abiotic environment. We therefore chose a second environment, hereafter referred to as “environment 2”. This consisted of growth in honeycomb plates at 28°C, 15 s of shaking 5 s before each OD measurement (600 nm, 400 μl of suspension per well, Bioscreen, Oy Growth Curves Ab Ltd, Helsinki, Finland), one measurement every 30 min for 3.5 days.

### 2.6 Statistical analyses

The OD after 3.5 days of growth was analyzed in two steps with two linear mixed models. The following model was used to analyze the effect of the evolution on strain and community phenotypes:

$$Y_{\text{ijkl}}=\mu +\alpha_{i}+\beta_{j}+\gamma_{k}+{\left(\alpha \beta \right)}_{\mathrm{ij}}+{\left(\alpha \gamma \right)}_{\mathrm{ik}}+{\left(\beta \gamma \right)}_{\mathrm{jk}}+{\left(\alpha \beta \gamma \right)}_{\mathrm{ijk}}+I_{l}+E_{\text{ijkl}}$$

*Y*_*ijkl*_ is the OD of the biological entity *i* (three levels: strain 1, strain 2, community), of identity *l* (24 levels: strain or community identity), of evolutionary history *j* (three levels: ancestor, evolved in isolation, evolved in community), and in environment *k* (two levels: environment 1, environment 2). *μ* is the intercept, *α*_*i*_ is the effect of the biological entity, *β*_*j*_ is the effect of the evolutionary history, *γ*_*k*_ is the effect of the environment. The interaction effects between (i) the biological entity and the evolutionary history (*αβ*)_*ij*_; (ii) the biological entity and the environment (*αγ*)_*ik*_; (iii) the evolutionary history and the environment (*βγ*)_*jk*_; (iv) the biological entity, the evolutionary history and the environment (*αβγ*)_*ijk*_ were also included in the model. *I*_*l*_ is the random effect of the strain or community identity, *E*_*ijkl*_ is the residual error.

A second linear mixed model was built to analyze the effect of the evolutionary history of the community members on the community phenotype:

$$Y_{\mathrm{jkl}}=\mu +\beta_{j}+\gamma_{k}+{\left(\beta \gamma \right)}_{\mathrm{jk}}+I_{l}+E_{\mathrm{jkl}}$$

*Y*_*jkl*_ is the OD of the community of identity *l* (8 levels: A to H), of evolutionary history *j* (five levels: ancestor, evolved in isolation, evolved in community, mixed 1, mixed 2), in environment *k* (two levels: environment 1, environment 2). *μ* is the intercept, *β*_*j*_ is the effect of the evolutionary history, *γ*_*k*_ is the effect of the environment, (*βγ*)_*jk*_ is the effect of the interaction between the evolutionary history and the environment. *I*_*l*_ is the random effect of the community identity, *E*_*jkl*_ is the residual error.

To go into the details of the responses for each community, the OD after 3.5 days was then analyzed with a linear model that included the identity of the individual as a fixed‐effect factor, as well as the evolutionary history and the interaction between the identity and the evolutionary history. One model was built for the strains and one for the communities in both environments. Then, the predictability of the community evolutionary response was analyzed by comparing the response of the community (i.e., change in OD during experimental evolution) to (i) the response of strain 1 evolved in community, (ii) the response of strain 2 evolved in community, (iii) the sum of the responses of strains 1 and 2 (which corresponds to the expected response under the hypothesis of an additivity of the individual responses, i.e., the absence of evolution of interspecific interactions). The mean responses and the corresponding 95% confidence intervals were obtained by bootstrapping (1000 iterations of the calculation of the response from randomly sampled values with replacement).

All the analyses were performed with R software version 3.6.3 with lmerTest package for linear mixed models (Kuznetsova et al., 2017), car package for type II analyses of variance (Fox & Weisberg, 2019), and emmeans package for pairwise comparisons (Lenth, 2021).

## 3 RESULTS

### 3.1 The strains' responses are driven by their initial productivity in monoculture

**FIGURE 2:**
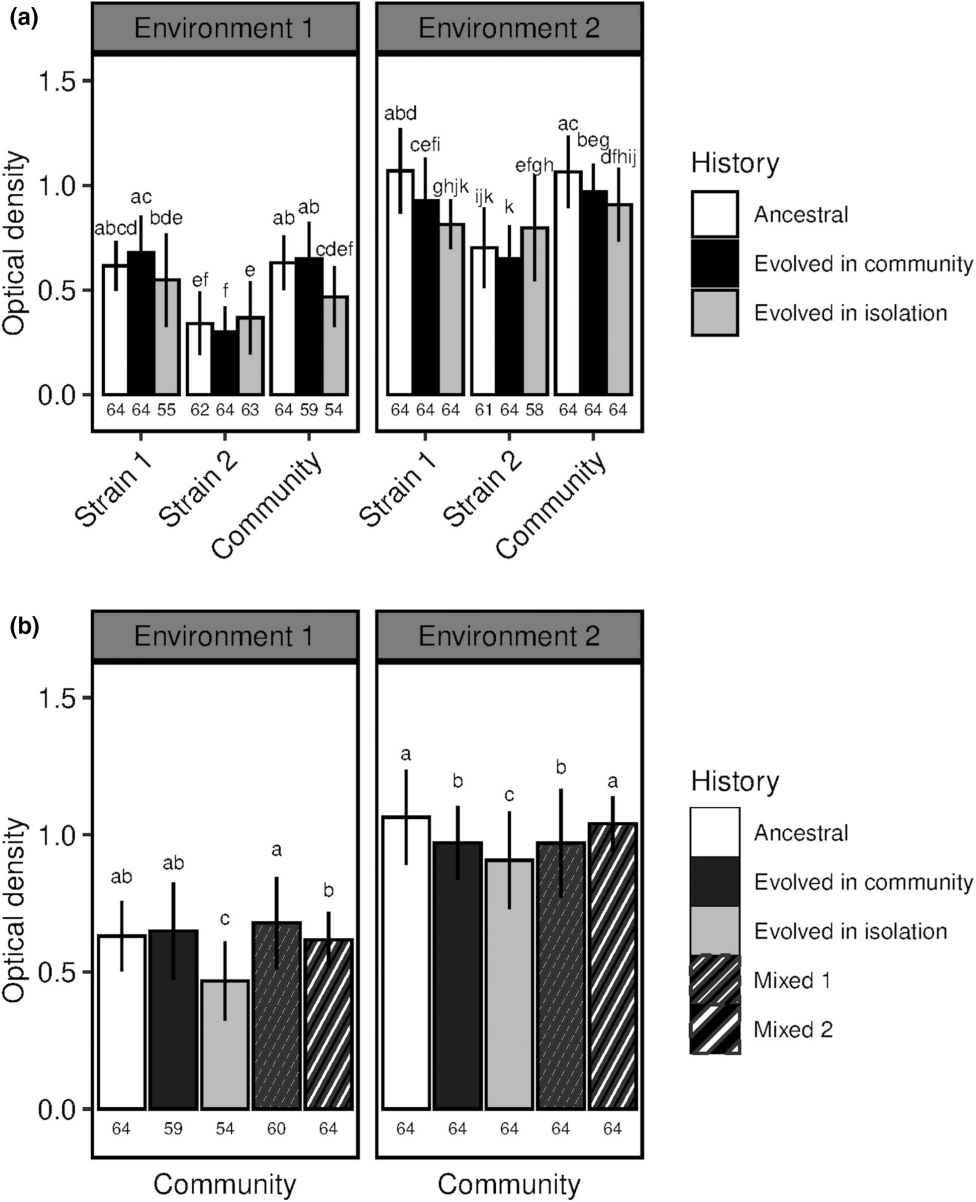
Optical density depending on the evolutionary history and the environment. (a) Ancestral and evolved strains and communities. (b) Communities depending on the evolutionary history of their members. Environment 1: identical growth conditions to the experimental evolution; Environment 2: different growth conditions from the experimental evolution. Strain 1 is the most productive of the two strains in a given community (highest OD) and strain 2 is the least productive of the two strains (lowest OD). Community refers to the co‐culture of strain 1 and strain 2. In mixed community 1, the strain 1 evolved in community was grown with the ancestral strain 2. In mixed community 2, the ancestral strain 1 was grown with the strain 2 evolved in community. Different letters represent significant differences in OD within a given environment (α = 0.05). Mean values are given ± SD. Sample sizes are given at the bottom of the graphs.

The effect of the evolutionary history on optical density (OD) depended on the biological entity, i.e., whether the considered phenotype was this of the community or of the community members, and it also depended on the abiotic environment (biological entity*history*environment: 𝜒^2^ = 48; *p*_df = 4_ = 1.0 × 10^−9^; Table 2). Strain 1 and strain 2, the initially most and least productive strain, respectively, responded differently to the evolution in environment 1. The OD of strain 1 when evolved in community tended to be higher than that of strain 1 as an ancestor and was higher than strain 1 evolved in isolation (respectively, 0.68 ± 0.18, 0.62 ± 0.12, 0.55 ± 0.22; Figure 2a). On the contrary, strain 2 showed a lower OD when evolved in community as compared to evolved in isolation (0.30 ± 0.12 and 0.37 ± 0.18, respectively). The OD of the ancestral strain 2 was 0.34 ± 0.15, which was not significantly different from the two other treatments.

**TABLE 2:** Analysis of variance (ANOVA) of the optical density (OD) of the communities and community members.

	Df	Chi‐squared	*p*
Biological entity	2	19.3	6.38 × 10^−5^
History	2	104	<2.2 × 10^−16^
Environment	1	2817	<2.2 × 10^−16^
Biological entity * History	4	193	<2.2 × 10^−16^
Biological entity * Environment	2	19.3	6.32 × 10^−5^
History * Environment	2	46.5	7.95 × 10^−11^
Biological entity * History * Environment	4	47.9	1.01 × 10^−9^
		*R* ^2^ = 0.85	

*Note*: The effect of the biological entity (strain 1, strain 2, community), the history (ancestors, evolved in community, evolved in isolation), the environment (1, 2), and their interactions on OD were estimated with a linear mixed model including the identity of the strain or community as a random effect factor. The conditional *R* ^2^ is presented (i.e., variance explained by both fixed and random effect factors; the marginal *R* ^2^—fixed‐effect factors only—was 0.63).

### 3.2 Community response is driven by the most productive strain in monoculture

In environment 1, the OD of the communities composed of strains that evolved together was not significantly different from the OD of the ancestral communities (respectively, 0.65 ± 0.18 and 0.63 ± 0.13; Figure 2a). But, it was higher than the OD of the communities in which the members evolved in isolation (0.47 ± 0.15) suggesting that the evolution in community (i.e., co‐culture) did not produce the same outcome than evolution in isolation. However, the communities composed of strains that evolved together produced the same phenotype as mixed communities (one ancestral and one evolved strain, Figure 2b).

The OD of the community was not different from the OD of strain 1 whatever the evolutionary history (respectively, 0.61 ± 0.16 and 0.62 ± 0.18 on average; Figure 2a). Also, the response of the community to the evolutionary history was similar to this of strain 1 (i.e., trend to increase in OD with evolution in community as compared to the ancestor and trend to decrease in OD with evolution in isolation; Figure 2a, environment 1). Thus, community phenotype seemed to be driven by strain 1.

### 3.3 Community response involves an evolution of the interactions in half of the cases

**FIGURE 3:**
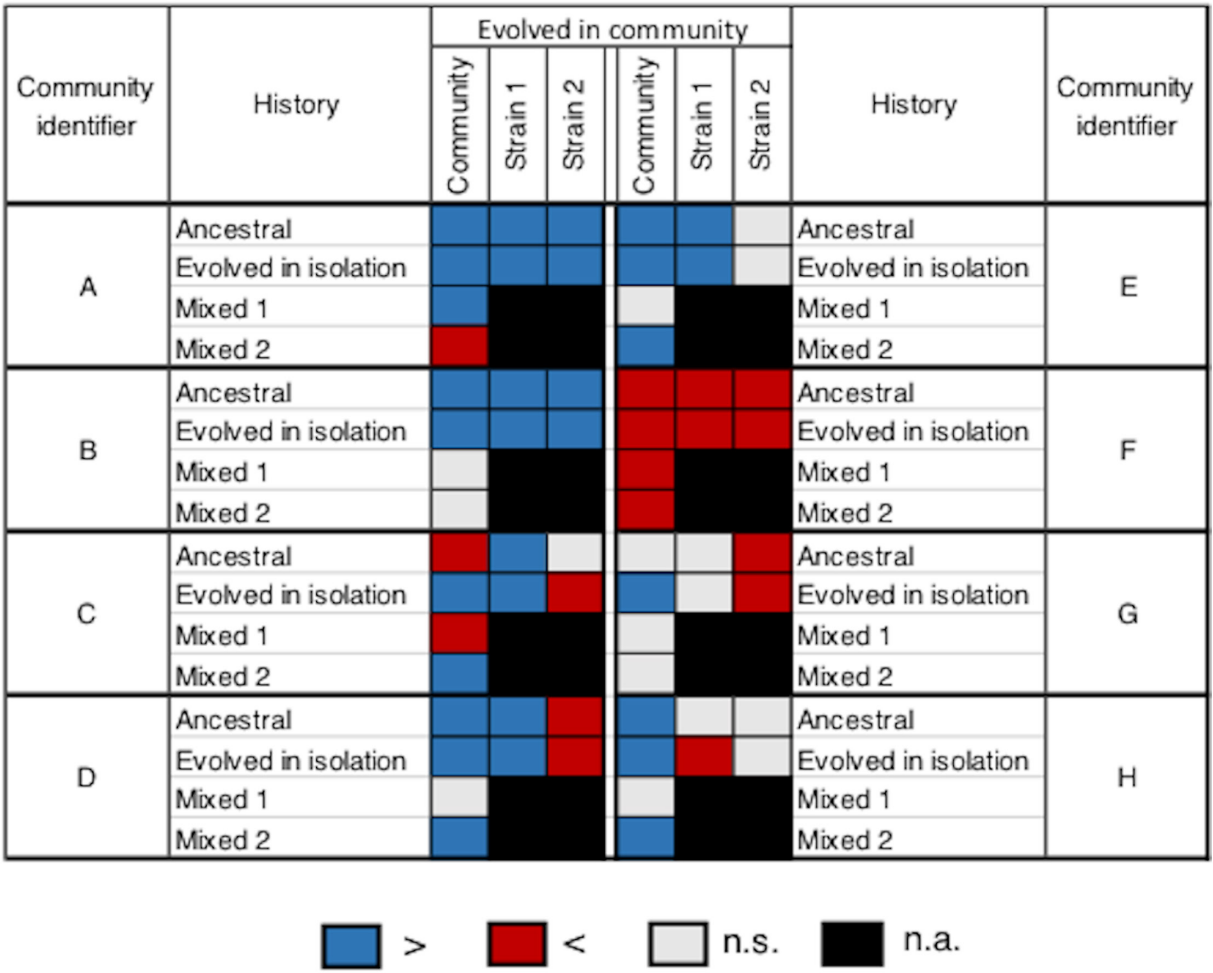
Effect of an evolution in community on optical density in environment 1 depending on the community. The OD of a community composed of strains that evolved together (in columns) is compared with the OD of a community including ancestral strains, strains evolved in isolation, or one ancestral strain and one strain evolved in community (mixed 1 and mixed 2) (in rows). The OD of strains 1 and 2 evolved in community (in columns) is compared with the OD of the corresponding strain as an ancestor or evolved in isolation (in rows). Blue: significantly higher. Red: significantly lower. Light gray: no significant difference (α = 0.05). Black: not applicable.

For all of the studied communities, there was a significant difference in OD between the evolved in community and evolved in isolation treatments (Figure 3), which highlighted the importance of the interactions in the evolution of community phenotype. This difference was in favor of the evolved in community treatment in seven of the eight communities (higher OD than evolved in isolation, Figure 3). One evolved community showed no difference in OD as compared to the ancestral community (community G; Figure 3). Three communities showed differences in OD with the communities of all other evolutionary histories (communities A, C, and F). It indicated that, in these cases, the only way to obtain the evolved community phenotype was through the presence of the two strains in their evolved community version. The four remaining communities (B, D, E, and H) showed no difference in OD as compared to at least the mixed community 1 (Figure 3) highlighting the role of strain 1 in the expression of the evolved community phenotype in these cases.

**FIGURE 4:**
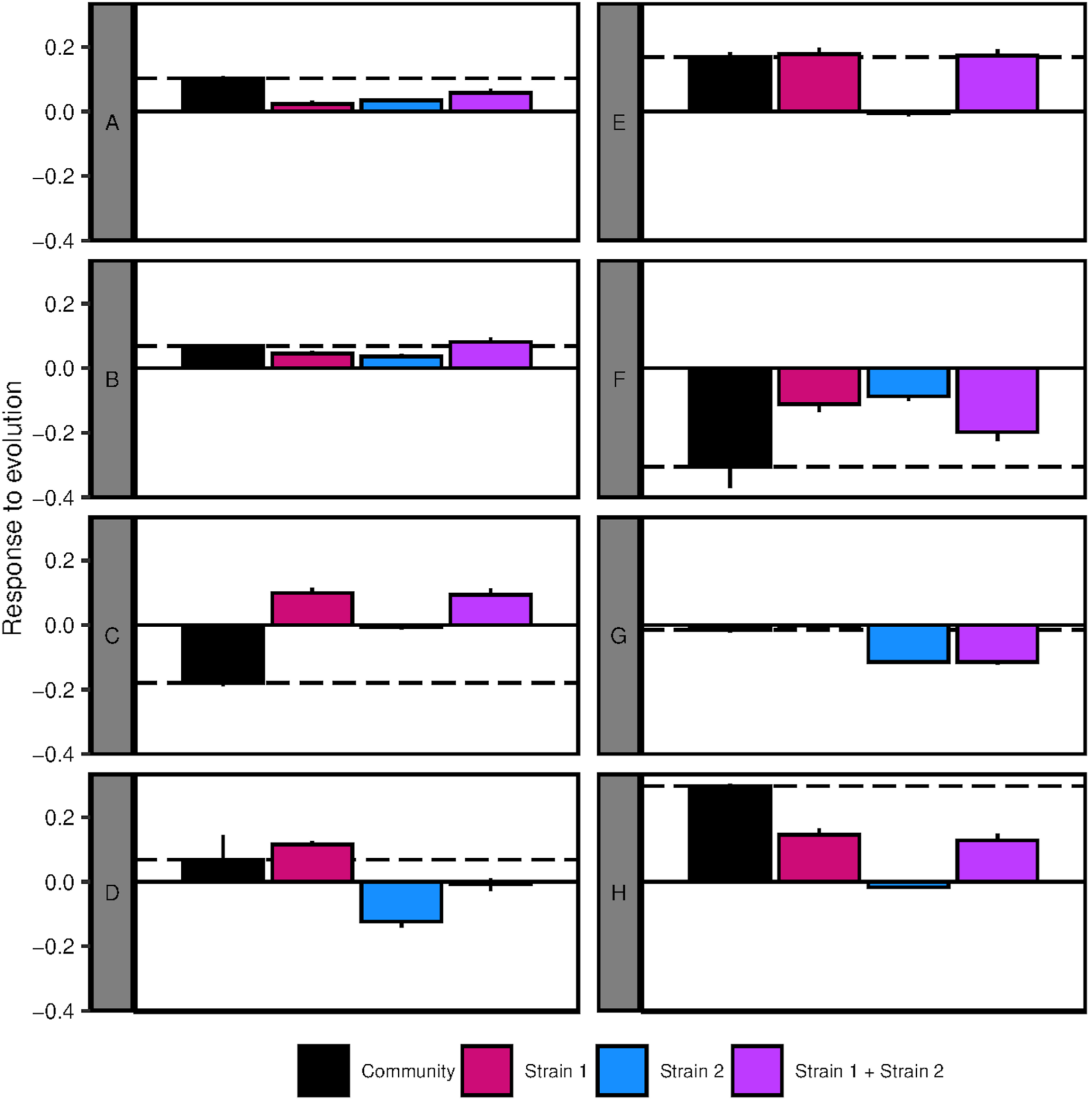
Predictability of the evolutionary response of the community. The observed evolutionary responses of the community, strain 1, and strain 2 in environment 1 were expressed as the difference in optical density between the treatment evolved in community and the corresponding ancestor (i.e., ancestral community or ancestral strain 1 or ancestral strain 2 in environment 1). “Strain 1 + Strain 2” refers to the expected response to evolution under the hypothesis of additivity of the individual responses, it was obtained by summing the observed responses of strain 1 and strain 2. Bars represent 95% CI. On each graph, the black dashed line represents the mean value of the response of the community. Mean values and 95% CI were obtained by bootstrapping.

The evolutionary response of four of the communities was predictable neither from the responses of the community members nor from the expected response under the hypothesis of additivity of the individual responses, i.e., an absence of evolution of the interspecific interactions (communities A, C, F, and H; Figure 4). It suggested that the evolutionary response involved an evolution of the interactions. In communities D and E, the community response was predictable from the response of strain 1, and in community B, it was predictable from the sum of the responses of the two strains (Figure 4). Thus, we did not evidence an evolution of the interspecific interactions in these communities.

### 3.4 The abiotic environment influences the evolutionary responses

In environment 2, where the conditions differed from these of the experimental evolution, strain 2 showed a similar response to the evolutionary history than in environment 1 (Figure 2a). On the contrary, the responses of strain 1 and of the community changed: the highest OD was observed for the ancestors followed by evolved in community and by evolved in isolation treatments. The expression of the “evolved phenotype” thus depended on the abiotic environment. As in environment 1, community phenotype and community response to the evolutionary history were similar to strain 1 (Figure 2a). The OD of the mixed community 1 was similar to this of the community in which the strains evolved together (respectively, 0.97 ± 0.20 and 0.97 ± 0.13) whereas mixed community 2 showed a higher OD that did not differ from that of the ancestral community (Figure 2b). This clearly highlighted the influence of strain 1 on community phenotype.

**FIGURE 5:**
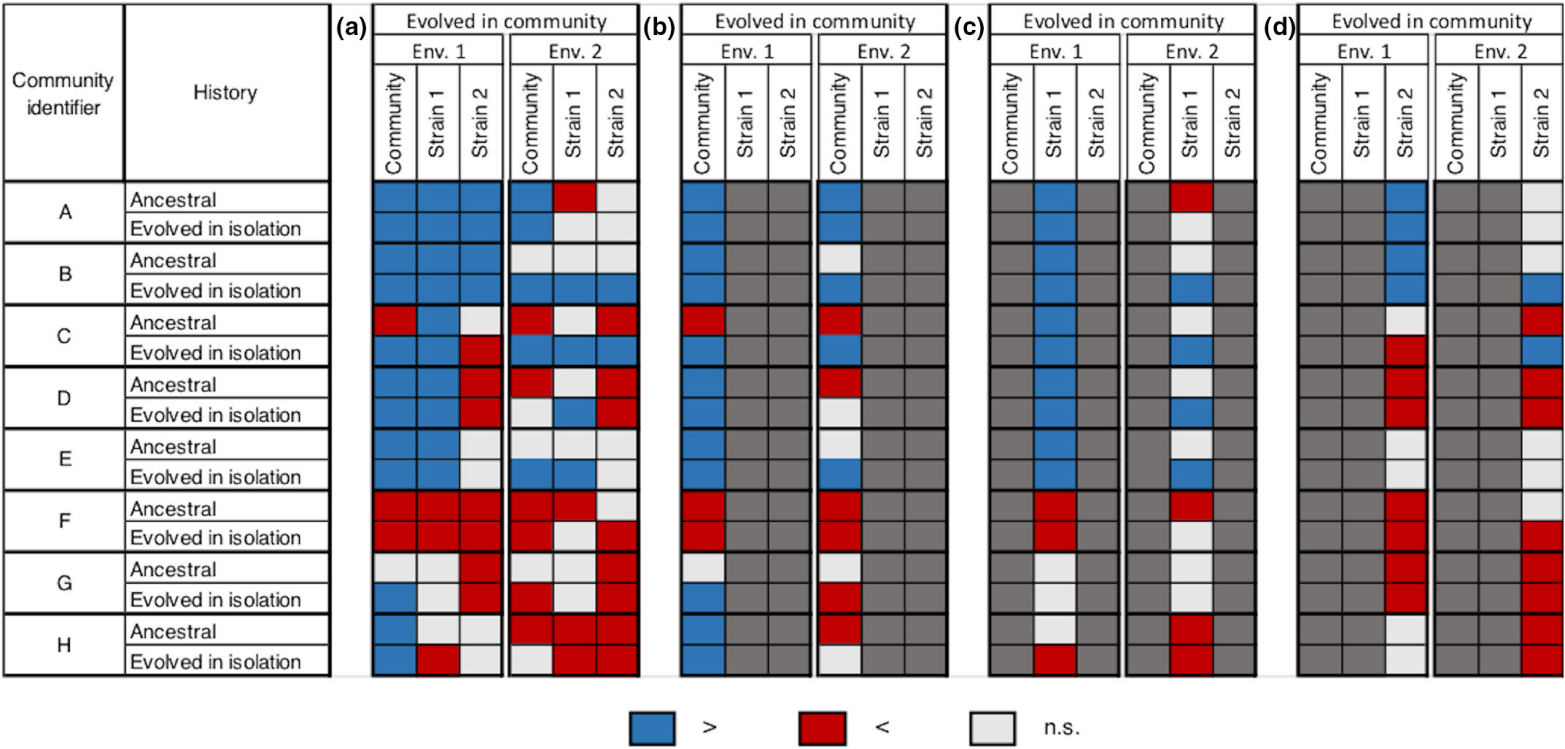
Effect of the environment on the expression of the evolved phenotype. The OD of a community composed of strains that evolved together (in columns) is compared with the OD of a community including ancestral strains or strains that evolved in isolation (in rows). Moreover, the OD of strains 1 and 2 evolved in community (in columns) is compared with the OD of the corresponding strains as ancestors or evolved in isolation (in rows). The results are presented for both environments. Environment 1: identical growth conditions to the experimental evolution; Environment 2: different growth conditions from the experimental evolution. Blue: significantly higher. Red: significantly lower. Light gray: no significant difference (α = 0.05). The overall results are shown on panel a, and panels b, c, and d show the results of the community, strain 1, and strain 2, respectively. On those panels, only the comparisons of interest are shown, and the others are shaded in dark gray for readability.

Whether the detected response to evolution (i.e., positive or negative change in OD) in environment 1 was also observed in environment 2 depended on the considered strain or community (Figure 5a). The phenotypic change in response to evolution in the evolved community (i.e., change in OD as compared to the ancestral community or to the community with evolved in isolation members) was maintained in environment 2 for three communities over eight (A, C, and F; Figure 5b). When a strain that evolved in community showed a significant increase in OD as compared to the ancestor, this pattern was always lost when the environment changed (Figure 5c,d). On the contrary, when a strain that evolved in community showed a significant decrease in OD as compared to the ancestor, this pattern was maintained in environment 2 in three cases over four. The changes in OD in a strain that evolved in community as compared to the corresponding strain that evolved in isolation were maintained in environment 2 in nine cases over 13 (Figure 5c,d).

## 4 DISCUSSION

We showed that the evolution of the strains in a community was influenced by interspecific interactions. Indeed, evolution in isolation did not produce the same phenotype as evolution in community (Figure 2a). These results are in accordance with an increasing body of literature that highlights the effect of the biotic context, i.e., of the evolution within a community, on the evolutionary response, and on the fitness of the community members (Fiegna et al., 2015; Hansen et al., 2007; Jousset et al., 2016; Scheuerl et al., 2020). The characterization of the community members on the basis of their productivity before experimental evolution allowed a good explanation of their responses to evolution despite the fact that we grouped species from different genera under the entities “strain 1” and “strain 2” (model *R* ^2^ = 0.85; Table 2). To go further, we found that the most productive strain had a dominant role in explaining community phenotype and community response to evolution (Figure 2a). It was probably highly linked to the fact that the studied community phenotype was productivity but, it also suggested that the most productive strain in monoculture was also the dominant strain in the community as previously observed in two‐species communities (Meroz et al., 2021).

Beyond an effect at the individual level, our results indicated that the evolution of community phenotype, i.e., productivity, was influenced by evolutionary changes in interspecific interactions. Indeed, as in a previous study (Lawrence et al., 2012), the phenotype of the evolved community could not be obtained by reconstructing a community from strains that evolved in isolation (Figures 2 and 3). We observed an effect of the interactions on community evolutionary response in all of the communities that showed an evolution in their phenotypes, i.e., seven among the eight (Figure 3, except G). However, this effect of the interactions depended on the studied community and occurred in three different ways. Community phenotype evolved through (i) an evolutionary response of one strain conditionally to the presence of the second strain without the evolution of the interaction (communities D and E), (ii) an evolutionary response of the two strains conditionally to their respective presence without the evolution of the interaction (B), (iii) an evolution of the interaction itself under the influence of one (H) or of the two strains (A, C, and F; Figures 3 and 4). Thus, the evolution of the community phenotype involved an evolution of the interactions in more than half of the cases. It suggested that the implication of the evolution of the interactions in the evolution of community phenotype is not rare in the experimental evolution of microbial communities. In another study (Williams & Lenton, 2007), a modeling approach showed that the responses of ecosystems to evolution under artificial selection would involve an evolution of the interspecific interactions in 4% of the cases when targeting an increase in a property and in 38% of the cases when targeting a decrease in a property (this could be modulated by specific experimental choices). More recently, it has been estimated that the evolution of the productivity of beech tree bacterial communities was explained by ecological sorting at 0.35%, by additive evolution at 17.7%, and by the evolution of the interspecific interactions at 14.3% (Fiegna et al., 2015). It is not straightforward to estimate the importance of the interspecific interactions in community evolutionary dynamics as their role seems to be highly dependent on the studied community but, together, these results suggest that it is relevant to consider the evolution of the interactions when studying community dynamics, at least in laboratory experiments.

In the communities in which an evolution of the interspecific interactions was detected, the change in community productivity was higher than expected, but the direction of this change was community‐dependent. The response to evolution when the interactions evolved (i.e., in communities A, C, F, and H) gave rise to a mean change in the productivity of 35 ± 13%, i.e., +15 ± 7% as compared to what was expected from the individual responses. However, in two communities over four (C and F) this change was negative (i.e., the productivity of the evolved community was lower than this of the ancestral community), and in one case, it occurred whereas the sum of the individual responses was positive (C; Figure 4). In the other studies that reported an evolution of interactions, the effect was to enhance community productivity (Fiegna et al., 2015; Hansen et al., 2007; Lawrence et al., 2012). Furthermore, some authors showed a reduction in the negative interactions and the evolution towards positive ones (Lawrence et al., 2012). In our study, we did not characterize the interactions, but we can hypothesize that different types of interactions (i.e., positive or negative) led to different responses of the community phenotype to the evolution of the interactions.

The influence of the abiotic environment on the evolutionary responses of the communities and community members was community‐dependent. For three of the four communities in which an evolution of the interactions was detected, the response to evolution was consistently observed in the two environments (communities A, C, and F; Figure 5b) contrary to what was observed for the strains composing these communities (Figure 5c,d). It suggested that the evolutionary responses of the strains involved an adaptation to the abiotic component (so that the response is not consistently observed when changing the environment), but that the expression of the “evolved” interaction did not rely on an adaptation to the abiotic component or relied on an adaptation to a condition that is found in the two environments (Hillesland & Stahl, 2010). Previous studies have shown the importance of resources on the outcome of the evolution of interactions (Lawrence et al., 2012; Rivett et al., 2016). As the same culture medium was used in the two environments in our experiment, it could suggest that the evolution of the interactions implied modifications in resource sharing.

Our results also suggested that the evolution in community often promoted an adaptation of the strains to the abiotic component, especially in strain 1 (Figure 5c,d). This is not expected since the theory predicts that there are trade‐offs between the adaptation to the abiotic and to the biotic components (Barraclough, 2015; Lawrence et al., 2012) and, that biotic forces are dominant over abiotic forces in driving species evolution (Red Queen hypothesis; Brockhurst et al., 2014). Thus, it is expected that strains that evolved in isolation would show a better adaptation to the abiotic environment than strains that evolved in community. It has been observed experimentally (Castledine et al., 2020; Lawrence et al., 2012) but seemed to be strain‐dependent. Our results suggested that the interspecific interactions could have promoted evolutionary responses to the abiotic conditions, which can occur through competition for example (Barraclough, 2015). These results may be linked to the structure of the environment. Indeed, it has been suggested that in homogeneous environments, the evolution would act through the selection of traits that are directly beneficial for the carrier species (Gorter et al., 2020). Thus, the evolutionary response of a strain to the presence of another strain could be an adaptation to the abiotic conditions, which could have a direct and positive effect on the strain fitness.

In this study, we aimed at investigating the importance of the evolution of the interactions in community evolution. There was evidence for an evolution of the interactions in half of the studied communities. Moreover, even when they did not evolve themselves, the interactions influenced the evolution of both community phenotype and community members' phenotype. To go further, our results suggested that the communities in which an evolution of the interspecific interactions was detected were also the most robust to environmental change regarding the expression of community phenotype. This is of particular interest in the field of the artificial selection at the community level and its possible applications.

## AUTHOR CONTRIBUTIONS

**Tiffany Raynaud:** Conceptualization (equal); data curation (lead); formal analysis (lead); investigation (lead); software (lead); visualization (lead); writing – original draft (lead). **Manuel Blouin:** Conceptualization (equal); funding acquisition (lead); supervision (equal); validation (equal); writing – review and editing (equal). **Marion Devers‐Lamrani:** Investigation (supporting); resources (equal); writing – review and editing (equal). **Dominique Garmyn:** Investigation (supporting); resources (equal); writing – review and editing (equal). **Aymé Spor:** Conceptualization (equal); formal analysis (supporting); software (supporting); supervision (equal); validation (equal); writing – review and editing (equal).

## CONFLICT OF INTEREST

The authors have no conflict of interest to declare.

## Data Availability

All the data and codes that support the results of this study are available in Zenodo (https://doi.org/10.5281/zenodo.7189920).
